# Multimodal Diagnostics of Microrheologic Alterations in Blood of Coronary Heart Disease and Diabetic Patients

**DOI:** 10.3390/diagnostics11010076

**Published:** 2021-01-06

**Authors:** Anastasia Maslianitsyna, Petr Ermolinskiy, Andrei Lugovtsov, Alexandra Pigurenko, Maria Sasonko, Yury Gurfinkel, Alexander Priezzhev

**Affiliations:** 1 Physics Department, Lomonosov Moscow State University, 119991 Moscow, Russia; peter.ermolinskiy@biomedphotonics.ru (P.E.); anlug@biomedphotonics.ru (A.L.); avp@biomedphotonics.ru (A.P.); 2 International Laser Center, Lomonosov Moscow State University, 119991 Moscow, Russia; 3 Faculty of Fundamental Medicine, Lomonosov Moscow State University, 119991 Moscow, Russia; pigurenko-alexandra-101-16-17@yandex.ru; 4 Research Clinical Center of JSC “Russian Railways”, 123567 Moscow, Russia; msasonko@yandex.ru; 5 Medical Research and Educational Center, Lomonosov Moscow State University, 119991 Moscow, Russia; yugurf@yandex.ru

**Keywords:** blood rheology, red blood cell aggregation, laser tweezers, laser aggregometry, digital capillaroscopy, coronary heart disease, diabetes mellitus

## Abstract

Coronary heart disease (CHD) has serious implications for human health and needs to be diagnosed as early as possible. In this article in vivo and in vitro optical methods are used to study blood properties related to the aggregation of red blood cells in patients with CHD and comorbidities such as type 2 diabetes mellitus (T2DM). The results show not only a significant difference of the aggregation in patients compared to healthy people, but also a correspondence between in vivo and in vitro parameters. Red blood cells aggregate in CHD patients faster and more numerously; in particular the aggregation index increases by 20 ± 7%. The presence of T2DM also significantly elevates aggregation in CHD patients. This work demonstrates multimodal diagnostics and monitoring of patients with socially significant pathologies.

## 1. Introduction

Blood flow and circulation inside vessels in vivo are defined by many interconnected parameters, such as blood viscosity, hematocrit, and plasma protein composition, so to properly study them a complex approach is required [1]. There are many methods allowing for in vivo and in vitro measurements of different blood properties [1,2].

The main focus of this study is the aggregation of red blood cells (RBCs), which significantly influences the viscosity of blood [3]. RBC aggregation is the reversible process of the formation of linear and more complex structures of RBCs. It promotes the formation of peripheral cell-poor fluid layer that lowers the hydrodynamic vessel resistance to blood flow [3]. The process of in vitro as well as in vivo RBC aggregation can be described in several ways: by the number of cells aggregating during a given time interval, by how many aggregates are observed, by how fast a couple of RBCs can form a doublet, etc. [3]. These parameters require specialized tools in order to be measured, so they are not used widely in clinical conditions. RBC aggregation depends on many internal and external factors. For example, blood plasma composition, temperature, RBC shapes, and the age, state of health of an individual, and his or her medicine intake determine in part the aggregation parameters of RBCs [3,4,5].

Many methods can be applied to studying RBCs, including micropipette aspiration [6], etc., but among all methods it would be worth highlighting the optical techniques as far as they have several advantages: non-invasiveness and lack of direct mechanical contact with the cells, the option to study both individual cells and their ensembles, and the possibility of in vivo and in vitro application [4,7]. The last point can pose a challenge in terms of comparing results for these different conditions—in vitro measurements require anticoagulants for stabilizing the blood samples and storing blood during sample preparation, which can influence the measured parameters, whereas in vivo methods allow for measuring a different set of parameters that may be difficult to correlate with those measured in vitro.

The aim of this work was to find correspondence between in vivo and in vitro optical methods by studying patients with cardiovascular and associated pathologies. Understanding the link between RBC aggregation and widespread cardiovascular diseases is vital to create new methods of diagnosis and treatment. In this article, we look at coronary heart disease (CHD) and type 2 diabetes mellitus (T2DM), which are known to have a significant effect on microvasculature [8].

## 2. Materials and Methods

### 2.1. Patients

The study enrolled 81 adults, including 25 healthy volunteers and 56 patients with CHD and arterial hypertension (Table 1). The patients with CHD were divided into two groups depending on the presence of T2DM. The first group included 42 CHD patients without T2DM, and second group included 14 CHD patients with T2DM. We did not find any statistically significant differences in the clinical background for these two groups of patients. The study was conducted during the period from July 2015 to July 2020.

Criteria for exclusion from the study were the following: chronic heart disease insufficiency, heart rhythm and conduction disorders, renal and hepatic insufficiency, type 1 diabetes mellitus, vascular or other pathology of the brain, or oncological diseases in the medical history.

The study included several procedures for the patients. The measurements of blood pressure and heart rate were conducted for each patient between 8:00 and 8:30 a.m. before their medicine intake on the day following their hospitalization in the cardiological unit. In addition to the standard medical check-up, all patients underwent a non-invasive study of microcirculatory parameters in the nail bed capillaries using the method of digital capillaroscopy. For the in vitro study, the blood was drawn from the patients’ cubital veins on an empty stomach, stabilized with EDTA K2 anticoagulant and was used for the experiments within the first three hours.

Twenty-five healthy volunteers (16 males and 9 females) had an average age 27.5 and body mass index (BMI) of 22.1, were non-smokers, and had not taken any medication. These people were divided into two control groups (*n* = 10 (4 males and 6 females) and 15 (12 males and 3 females)) that were studied independently at different time intervals with different parameters measured.

The study design was approved by the local ethics committee of medical research and educational center of M.V. Lomonosov Moscow State University, Moscow, Russia (protocol code: 1/19, date of approval: 18 February 2019). The experiment design took into account the latest recommendations for laboratories made by the international expert team for the standardization of hemorheological methods [9]. The patients and healthy volunteers participating in the research were informed on the purpose of the study and gave written informed consent in accordance with the Declaration of Helsinki.

### 2.2. Laser Aggregometry Method

The laser aggregometry method implemented in a microchip stirring type RheoScan aggregometer (RheoMedTech, Seoul, Korea) was used to measure the aggregation parameters of RBCs in vitro in whole blood samples [10]. Laser aggregometry is based on the diffused light scattering of a laser beam by the blood sample [11]. To perform the measurements 8 μL of whole blood were placed inside a flat reservoir and heated up to 37 °C. Then the sample was illuminated by a laser beam (633 nm, 2.5 mW), which was scattered by the RBCs and their aggregates mostly in the forward direction—the scattering particles were much larger than the light wavelength. The larger the size of the scattering particle the more intensity was scattered forward [12,13].

**Figure 1:**
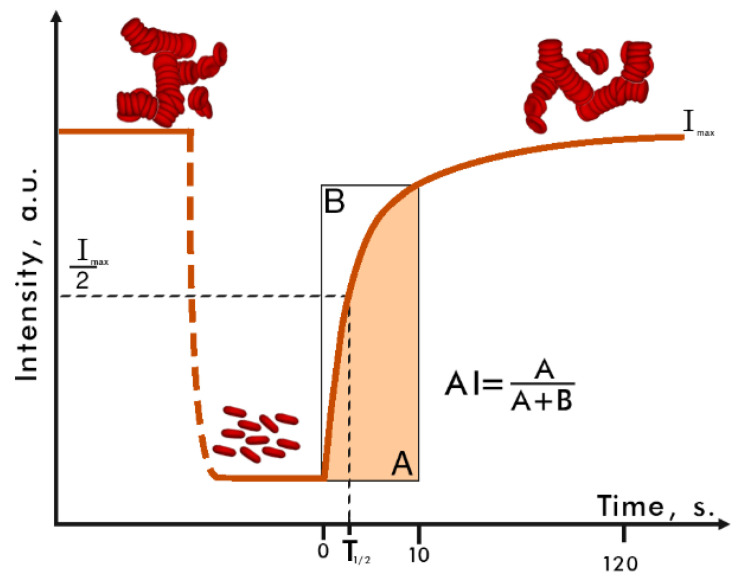
The kinetics of the RheoScan aggregometer output signal (intensity of light scattered forward as a function of time). The meaning of T_1/2_ and AI is indicated. ‘A’ is the area below the intensity curve inside the rectangle; ‘B’ is the area above the intensity curve inside the rectangle.

The time course of measured intensity of light scattered forward is presented in Figure 1. In the beginning of the measurement the RBCs were in a state of maximum aggregation and therefore the scattered intensity was also at its maximum. Then, a small magnetic bar inside the reservoir started stirring the sample, causing shear stress-induced destruction of the aggregates. After the stirring process (*t* = 0 in Figure 1), all aggregates were dispersed and none of the RBCs were in the aggregated state and therefore the scattered intensity was at its minimum; the cells themselves suffered no permanent damage from this. The stirring stopped and so the RBCs started to aggregate again, the average size of a scattering particle in the sample grew, and the scattered intensity increased. The aggregation process lasted approximately two minutes; after that the intensity reached its maximum, indicating that the cells were in a state of complete aggregation.

The aggregation kinetics were reflected in the dependence of the scattered intensity on time and several parameters were calculated based on it. Firstly, the characteristic time of aggregate formation (T_1/2_) characterized the time interval, during which the signal (scattered light intensity) reached half of the maximum value (see Figure 1). A smaller T_1/2_ indicated greater curve slope and faster aggregation of the cells. Secondly, the aggregation index (AI) characterized the fraction of cells aggregated during the first 10 s of measurement. It was calculated as a ratio between the area under the intensity curve to the total area above and below it (see Figure 1). Higher AI values corresponded to more numerous RBC aggregation in the sample.

### 2.3. Laser Tweezers

Laser tweezers (LT) are scientific tools that allow for the trapping and manipulation of microobjects (such as living cells, etc.) using a highly focused laser beam [14]. LT are essential instruments in single-cell studies. The physical principle of optical trapping is described in more detail in [15].

In our study, LT were used to measure the duration of the process of spontaneous aggregation of two individual RBCs [16]. Two beams from the Nd:YAG lasers (1064 nm, 200 mW) propagated through a system of lenses and polarizers and reached the dichroic mirror where they were split in two parts: The first one was directed to the aperture of the Olympus objective (×100, NA = 1.00, water immersion) and into the sample, and the second one to the photodetector in order to evaluate its power. One laser beam was always stationary whereas the other one could be moved by rotating the movable mirror—this allowed for two laser traps in the sample to be obtained: one stationary and one movable.

The measurements were carried out at room temperature (22 °C) in a highly diluted blood suspension inside a glass microcuvette with a 100 μm gap [17]. Patients’ autological platelet-poor plasma was used as the suspension medium. The plasma was acquired by centrifuging all the blood for 10 min at 170 g, removing the platelet-rich plasma, then centrifuging the platelet-rich plasma for 10 min at 3000 g and removing the buffy coat. Lastly, the RBCs were added to the plasma to achieve the final hematocrit of about 0.1%. Due to such a high dilution of the suspension the hematocrit did not affect the measurements.

**Figure 2:**
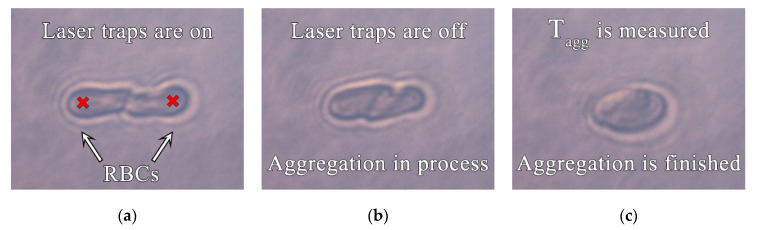
Three steps of T_agg_ measurement. Red crosses indicate the laser traps. (**a**) Two separate RBCs are brought together until the state of a “point contact”; (**b**,**c**) RBCs start to aggregate (overlap) after the laser beams are shut off.

In LT the trapped RBCs oriented themselves vertically, so the cells were observed edge-on [18]. The time of doublet aggregate formation T_agg_ was measured by orienting two trapped RBCs parallel to each other and creating a single point of contact between their membranes using the movable laser trap (see Figure 2a) [16]. Then, the laser traps were disabled and the time of the doublet formation as a result of spontaneous aggregation was measured (see Figure 2b,c). Smaller values of T_agg_ corresponded to faster aggregate formation.

For the interpretation of the results, it needs to be emphasized that the T_agg_ parameter corresponds to the initial stage of the aggregation process, whereas the laser aggregometry parameters (T_1/2_ and AI) represent the whole aggregation process, including the later formation of complex 3D aggregate structures.

### 2.4. Digital Capillaroscopy

Digital capillaroscopy was used to evaluate capillary blood flow parameters in vivo. Using the Kapillaroskan-1 device (AET, Moscow, Russia) a quantitative assessment of the blood flow characteristics was carried out in the nail bed capillaries. This method is described in more detail in [19,20]. Several nail bed capillaries of each patient were recorded at the high frame rate and then used to assess the average capillary blood flow velocity (CBV), which was calculated by frame-by-frame analysis.

Before the start of the measurements, the temperature of the skin of the studied finger was measured using an AND DT-635 skin thermometer (A&D, Tokyo, Japan). On average, the temperature of the skin of the finger in the patients included in the study was 33.6 ± 1.3 °C. There were no statistically significant differences between the groups considering the finger temperature.

### 2.5. Statistical Analysis

For each blood sample, the parameters AI T_1/2_, and T_agg_ were measured 5 times. The calculation of the CBV and the presence of aggregates for the DC method was carried out using original software that analyzed recordings of the nail bed capillaries. Videos of at least 6 good reading capillaries were used for calculations with a video duration of 3 to 5 s (at a recording rate of 100 frames per second, i.e., from 1800 to 3000 frames per patient). In the Results section the averaged values and the standard errors of the mean are presented. They were chosen over the standard deviations because they show the precision of the mean value. Statistical difference was calculated with a two-tailed Student *t*-test with unequal variance. The difference between the two values was considered statistically significant if the *p*-value according to the *t*-test was less than 0.05.

## 3. Results

### 3.1. Comparison of CHD Patients and Healthy Volunteers

The parameters assessed with the methods described above for all the CHD patients were different from the control values (Table 2). The first and second control group did not show statistically significant differences between themselves.

The values for control groups A and B did not show statistically significant differences for AI and T_1/2_ (see Table 2), which proves the consistency of the laser aggregometry method. Unfortunately, other parameters (T_agg_ and CBV) could not be measured for both control groups, but they were consistent with our previous work [11]. Control group A was studied more recently and had a greater number of subjects than group B, so we used it in the analysis.

In CHD patients compared to the control group A, AI was higher by 20 ± 7% (*p* < 0.05), meaning more numerous aggregation, T_1/2_ was lower by 14 ± 9% (*p* < 0.05), meaning faster aggregation, and T_agg_ was lower by 27 ± 7% (*p* < 0.05), showing faster doublet formation. As for CBV, it was smaller than the control, but significant only at *p* = 0.1 level due to high variation from person to person.

The CHD patients were divided into two groups by the presence of T2DM and they showed significant (*p* < 0.05) differences in some parameters (Table 3).

### 3.2. Digital Capillaroscopy Results Matched with In Vitro Parameters

**Figure 3:**
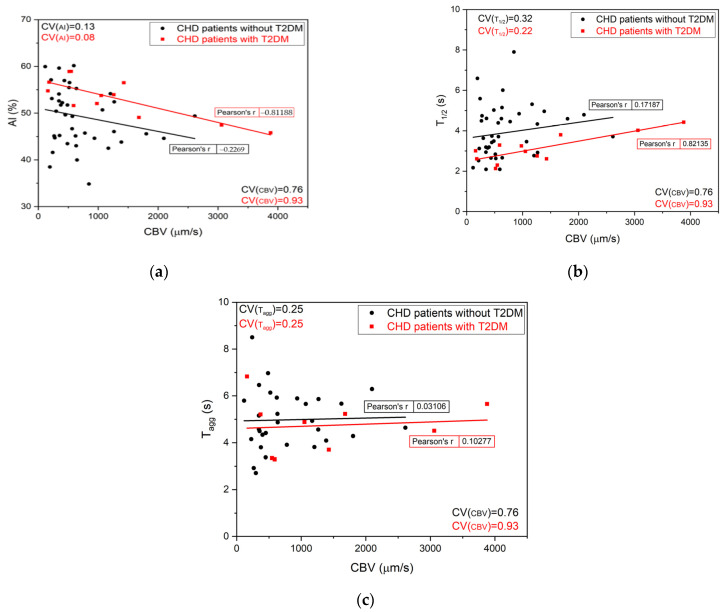
The individual values of AI (**a**), T_1/2_ (**b**), and T_agg_ (**c**) for groups of patients with and without T2DM versus the CBV. The linear fit and the Pearson’s r coefficient are shown. CHD—coronary heart disease; T2DM—type 2 diabetes mellitus; CBV—capillary blood velocity.

Figure 3a–c shows the RBC aggregation parameters plotted as functions of CBV and the Pearson’s r coefficient for each trend. AI for all the presented groups decreased with the increase of CBV, as indicated by the negative r. T_1/2_, on the other hand, increased and had positive r values. T_agg_ remained constant for the whole CBV range. These results show that for patients with high CBV the aggregation process in vitro was weaker compared to the patients with low CBV: The aggregation was less numerous and the doublet formation took longer.

No statistically significant difference in aggregation was found between several patient subgroups, including the division by gender and smoking habits. AI weakly correlated with BMI (Pearson’s r = 0.29) and did not correlate with age (r = −0.05).

## 4. Discussion

In vitro and in vivo aggregation conditions are, of course, different due to the channel shape, presence, or absence of an endothelium layer, surrounding medium, etc. [21]. Moreover, in vivo aggregation in big vessels differs from the aggregation in capillaries [3]. In this study, we investigated in vivo RBC aggregation in nailfold capillaries, therefore excluding effects that can be observed in big vessels, such as axial migration of RBC, etc. [3]. Hemorheological parameters (including blood flow in capillaries) show alterations in pathophysiological processes in a complex way [22]. That is why our data could have provided contradictory results if we had not used criteria for exclusion (mentioned above in Section 2. Materials and Methods 2.1. Patients).

We found correlations between blood flow parameters measured in vitro and in vivo, as well as significant differences between the control group and CHD patients with and without T2DM. Firstly, the RBC aggregation in CHD patients was enhanced compared to the healthy volunteers (see Table 2). The increase in RBC aggregation has already been linked to cardiovascular diseases, including CHD and arterial hypertension using different techniques [23,24,25]. However, the relationship between aggregation and pathologies is yet to be established—article [26] suggests that aggregation and pathologies are not linked directly but rather share the same factors, such as obesity and cigarette smoking, among others. We found only a weak correlation for these factors and aggregation in CHD patients.

Secondly, the presence of T2DM had a significant effect on aggregation properties: Both AI and T_1/2_ were higher (*p* < 0.05) compared to the patients without it (see Table 3). Of course, the effect of T2DM on RBC aggregation is already established [27], but being able to detect it specifically for CHD patients opens many doors in terms of diagnostics and monitoring the discussed pathologies. Two other parameters (T_agg_ and CBV) did not show significant differences; this could be in part due to the methodology. Both LT and capillaroscopy study a limited number of RBCs, whereas laser aggregometry analyzes ensembles of tens of thousands. A high uncertainty in T_agg_ and CBV demonstrated a great variation of RBC properties for each individual patient, which could have been caused by his or her health status. It could also mean that the effect of elevated aggregation was clearer during the later stages of aggregate formation (3D aggregate structures) than in the initial doublet formation.

Higher CBV means lower friction in the vessels and therefore weaker RBC aggregation [2]. This was clearly manifested for patients with T2DM in Figure 3 by the negative correlation with AI (r = −0.81) and positive correlation of T_1/2_ (r = 0.82). For patients with a high CBV it was more likely that their RBC aggregation be more numerous and take less time for a large ensemble. T_agg_ measured with LT did not correlate with CBV for all groups.

Additionally, it is important to mention the differences of blood temperature during measurement of different in vitro parameters. RheoScan parameters (AI and T_1/2_) were measured at 37 °C, whereas the LT parameter (T_agg_) was measured at room temperature at 22 °C. Because the aggregation depends on the temperature, these different conditions might have influenced the obtained data [3,28]. For example, RheoScan parameters (AI and T_1/2_) greatly depend on temperature [28]. However, in our previous work [29] it was shown that for LT measurements the temperature-dependent change of the RBC aggregation was nearly absent for the temperatures of 20 °C and 38 °C. This means that we could compare all in vitro parameters between each other as if they were measured at the same temperatures.

The novelty of the presented work consists of a complex analysis of in vitro and in vivo parameters for different pathologies. The results of studies performed by alternative methods do not contradict our conclusions and show increased aggregation of RBCs in patients with CHD (including various complications) compared to healthy donors [3,30,31,32].

One of the limitations of the study is the small number of patients with both CHD and T2DM; in the future, we plan to increase this number. This will allow for grouping the patients by specific medication used, such as antiaggregants and anticoagulants. Another point that can be improved is the observation of additional factors that influence the blood flow, for example plasma components [3,33]. In addition, BMI and other factors influence platelet activation and aggregation [34], which can indirectly affect the aggregation of RBCs; this was not accounted for in this article.

## 5. Conclusions

In this work, the aggregation of red blood cells was studied using different optical in vivo and in vitro measurement techniques. The aggregation for patients with coronary heart disease was statistically significantly enhanced compared to the control group. In vivo and in vitro methods yielded correlated results: The faster the cells moved in the capillaries, the less cells aggregated in vitro. Type 2 diabetes mellitus had an additional significant effect on the aggregation properties of coronary heart disease patients. These findings are prominent for diagnosing and monitoring the state of patients with pathologies that affect blood properties.

## Figures and Tables

**Table 1:** The clinical backgrounds for each group of patients, mean ± standard deviation or number (%).

Parameter	Overall Patient Data (*n* = 56)	Patients with CHD and without T2DM (*n* = 42)	Patients with CHD and T2DM (*n* = 14)
Number (percentage) of males	38 (68%)	29 (69%)	9 (64%)
Number (percentage) of females	18 (32%)	13 (31%)	5 (36%)
Mean age (range), years	69.2 (51–92)	70.5 (51–92)	65.3 (52–81)
Number (percentage) of smokers	10 (18%)	6 (14%)	4 (29%)
Body mass index, kg/m^2^	29 ± 5	28 ± 5	31 ± 5
Systolic blood pressure, mm Hg	139 ± 27	142 ± 22	137 ± 20
Diastolic blood pressure, mm Hg	82 ± 12	82 ± 13	83 ± 9
Heart rate, bpm	71 ± 9	72 ± 9	69 ± 10
LV ejection fraction, %	57 ± 7	56 ± 6	58 ± 8
Previous myocardial infarction	22 (39%)	18 (43%)	4 (29%)
Angina pectoris	48 (86%)	36 (86%)	12 (86%)
Bypass grafts	5 (9%)	4 (9%)	1 (7%)
Stents	17 (30%)	12 (29%)	4 (31%)
Antiaggregants	39 (70%)	29 (69%)	10 (71%)
Anticoagulants	12 (23%)	10 (24%)	3 (21%)
Diuretics	28 (50%)	19 (45%)	9 (64%)

CHD—coronary heart disease; T2DM—type 2 diabetes mellitus; LV ejection fraction—left ventricular ejection fraction.

**Table 2:** The comparison of CHD patients and the control groups. The averaged values and the standard errors are presented. The *p*-value was calculated by a two-tailed *t*-test with unequal variance.

Parameter/Group	Control Group A (*n* = 15)	Control Group B (*n* = 10)	CHD Patients (*n* = 56)
AI, %	41.0 ± 1.3	44 ± 3	49.0 ± 1.2 *
T_1/2_, s	5.9 ± 0.4	5.6 ± 1.0	4.2 ± 0.4 *
T_agg_, s	6.6 ± 0.4	-	4.8 ± 0.2 *
CBV, mcm/s	-	1290 ± 230	850 ± 100

CHD—coronary heart disease; AI—aggregation index; CBV—capillary blood velocity; * *p* < 0.05 for control group A.

**Table 3:** The comparison of CHD patients with and without T2DM. The averaged values and the standard errors of the sample mean are presented. The *p*-value was calculated by a two-tailed *t*-test with unequal variance.

Parameter/Group	CHD Patients (*n* = 42)	CHD + T2DM Patients (*n* = 14)
AI, %	48.6 ± 1.0	53.0 ± 1.1 *
T_1/2_, s	4.1 ± 0.2	3.1 ± 0.2 *
T_agg_, s	5.0 ± 1.2	4.6 ± 0.4
CBV, mcm/s	750 ± 90	1150 ± 300

CHD—coronary heart disease; T2DM—type 2 diabetes mellitus; AI—aggregation index; CBV—capillary blood velocity; * *p* < 0.05.

## Data Availability

The data presented in this study are available on request from the corresponding author. The data are not publicly available due to the ethical and privacy issues.
